# Autoimmune Theories of Chronic Spontaneous Urticaria

**DOI:** 10.3389/fimmu.2019.00627

**Published:** 2019-03-29

**Authors:** Sonali J. Bracken, Soman Abraham, Amanda S. MacLeod

**Affiliations:** ^1^Department of Internal Medicine, Duke University School of Medicine, Durham, NC, United States; ^2^Department of Pathology, Duke University Medical Center, Durham, NC, United States; ^3^Department of Immunology, Duke University Medical Center, Durham, NC, United States; ^4^Department of Dermatology, Duke University Medical Center, Durham, NC, United States

**Keywords:** chronic spontaneous urticaria, chronic idiopathic urticaria, autoimmune urticaria, anti-IgE, anti-FcεR1, autoallergy, anti-TPO

## Abstract

Urticaria (hives) is a highly prevalent skin disorder that can occur with or without associated angioedema. Chronic spontaneous urticaria (CSU) is a condition which persists for more than 6 weeks in duration and occurs in the absence of an identifiable provoking factor. CSU results from pathogenic activation of mast cells and basophils, which gives rise to the release of proinflammatory mediators that support the generation of urticaria. Several theories have been put forth regarding the pathogenesis of CSU with much evidence pointing toward a potential autoimmune etiology in up to 50% of patients with this condition. In this review, we highlight the evidence surrounding the autoimmune pathogenesis of chronic urticaria including recent data which suggests that CSU may involve contributions from both immunoglobin G (IgG)-specific and immunoglobulin E (IgE)-specific autoantibodies against a vast array of antigens that can span beyond those found on the surface of mast cells and basophils.

## Introduction

**Figure 1:**
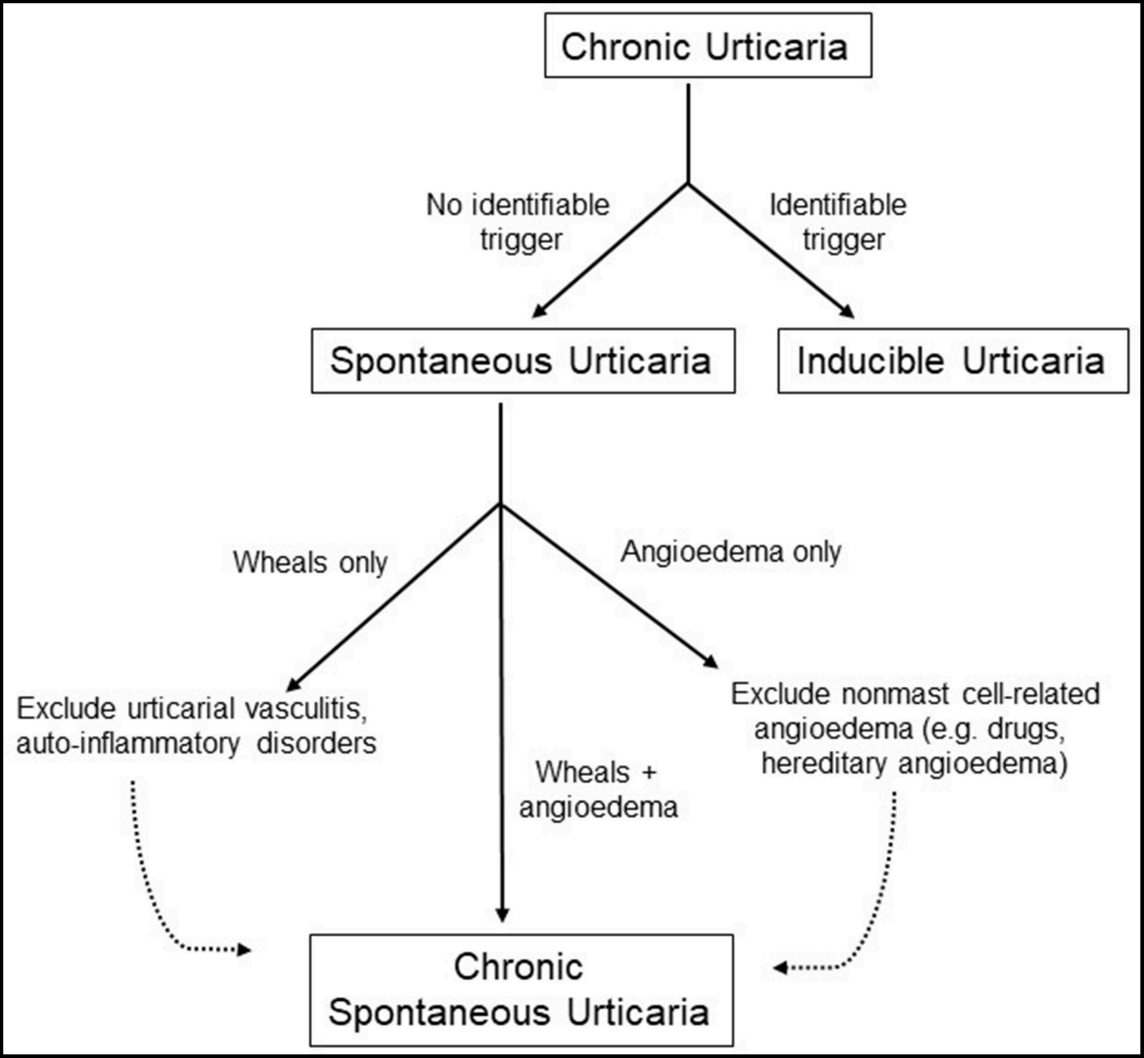
Diagnostic workup of chronic urticaria.

Urticaria, more commonly known as “hives”, is a prevalent disorder that affects between 15 and 25% of the population at some point during their lifetimes (1). The condition tends to be more common in adults than in children and in woman than in men with peak occurrence in the third to fifth decades of life. This condition is marked by the onset of pruritic “wheals,” which represent well-circumscribed areas of non-pitting edema with blanched centers and raised borders that involve only the superficial portions of the dermis and are seen in conjunction with surrounding erythema of the skin (2). Lesions can be as small as a few millimeters in diameter but can coalesce to form wheals as large as several centimeters wide. They often remit within 24 h since time of onset. Urticaria may be accompanied by the presence of angioedema, which is a similar process that occurs at submucosal surfaces of the upper respiratory and gastrointestinal tracts and deeper layers of the skin including subcutaneous tissue (3). Urticaria is classified as either acute or chronic depending on whether the onset of episodes lasts for less or >6 weeks in duration, respectively. In this review, we will focus specifically on the pathophysiology of chronic urticaria. Formerly referred to as chronic idiopathic urticaria, chronic spontaneous urticaria (CSU) refers to recurrent urticaria lasting more than 6 weeks that occur in the absence of an identifiable trigger. Urticaria that are incited by a well-defined eliciting factor (e.g., pressure, temperature, vibration) are referred to as inducible urticaria and will not be further discussed in this review. Prevalence of chronic urticaria is estimated to be anywhere from 0.5 to 5% in the general population but is not truly known, though incidence is thought to fall around 1.4% annually (4). Recent guidelines now include isolated idiopathic angioedema within the definition of CSU provided that other causes of angioedema, particularly those that are bradykinin mediated, have been excluded (5). Multiple studies have suggested that CSU may be an autoimmune condition in a substantial proportion of cases, but it is important to identify potential triggers of disease and exclude other differential conditions prior to making the diagnosis as outlined in Figure 1. In this article, we will discuss the pathophysiology of chronic urticaria and review the evidence surrounding its autoimmune etiology.

## Mast Cells and Basophils as Centralized Regulators of Chronic Spontaneous Urticaria

The pathophysiology of CSU is not well-understood, but it is clear that derangement of both mast cell and basophil activation and degranulation remains central to the process. Of these, mast cells are most widely accepted as the primary effectors of chronic urticaria. While other cell types including lymphocytes and polymononuclear cells (PMNs) have been observed within the inflammatory infiltrates of patients with CSU, it is well-established that histamine and other mast cell products are predominantly responsible for development of this condition [note that skin biopsies are seldom needed for the diagnosis but are occasionally useful to distinguish CSU from other inflammatory conditions such as urticarial vasculitis (5)]. The physical manifestation of urticaria can be attributed to the enhanced vascular permeability that results from the release of preformed mediators from mast cells (e.g., histamine, tryptase, leukotrienes) and their delayed generation of cytokines. Ongoing research continues to probe into the mechanisms by which mast cells are activated by blood-borne antigens, with recent data from murine *in vivo* studies suggesting that CD301b^+^ dermal dendritic cells (DCs) are actually first to sample antigen and then relay it to nearby mast cells through secreted microvesicles (6). However, the vast majority of cases of chronic urticaria are not triggered by any identifiable substance and are in fact idiopathic. In these cases, anaphylaxis does not occur though angioedema may be present.

**Figure 2:**
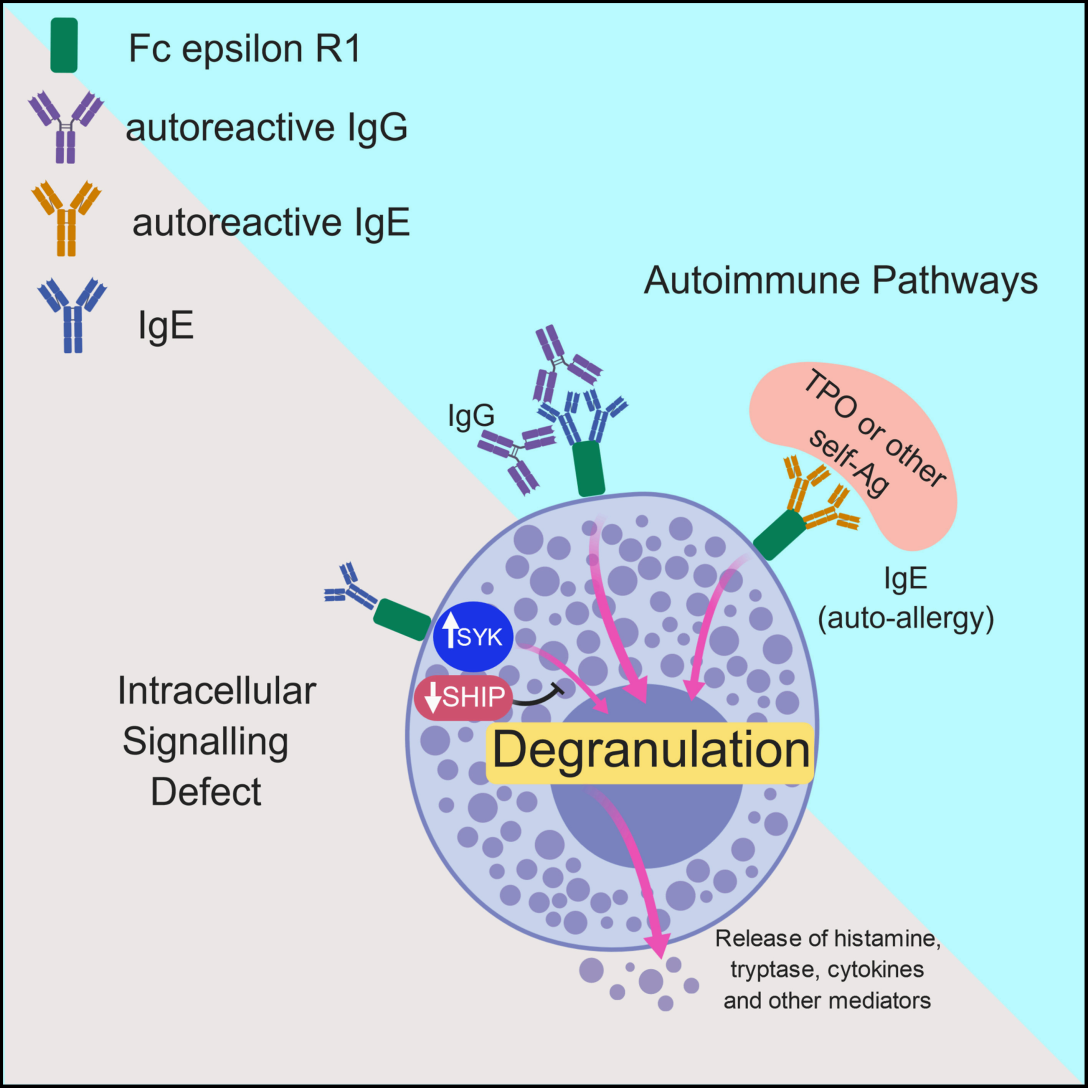
Model of the mechanisms underlying chronic urticaria. Pathologic activation of mast cells and basophils in patients with chronic spontaneous urticaria is thought to occur via two major mechanisms: intracellular signaling defects and autoimmune mechanisms. In the former, inappropriate activation of molecules such a spleen tyrosine kinase (SYK) or inhibition of negative regulators including the Src homology 2 (SH2)-containing inositol phosphatases (SHIP) promotes spontaneous degranulation of mast cells/basophils with subsequent release of histamine and other protein and lipid mediators. The more commonly accepted theory of pathogenesis in CSU includes antibody-mediated mast cell and basophil activation, which can occur via IgG- or IgE- mediated pathways. In the former, IgG molecules directed against the Fc portion of IgE or the FcεR1 promote spontaneous cellular degranulation. In patients with autoallergy, crosslinking of Fc epsilon R1 (FcεR1) via autoreactive IgE molecules directed against self-antigens such as thyroid peroxidase (TPO) promote mast cell/basophil degranulation.

Two major mechanisms have been put forward with regards to the pathogenesis of chronic urticaria. The first is not autoimmune in nature, but involves dysregulation of intracellular signaling pathways within mast cells and basophils that lead to defects in trafficking or function of these cells. The second involves the development of autoantibodies to FcεRIα or IgE on both mast cells and basophils and will be discussed in more detail in the following sections (7). Both of these mechanisms will be further explored here and are outlined in Figure 2.

## Dysregulation of Intracellular Signaling Pathways can Predispose to Pathologic Activation of Mast Cells and Basophils

Activation of the high-affinity IgE receptor, FcεR1, is an important step in the development of allergic responses and in the development of urticaria. This receptor is composed of an α-, β-, and two γ subunits (8). Whereas the α-subunit binds to the Cε3 constant region of the IgE molecule, the β-, and γ- subunits contain cell immunoreceptor tyrosine-based activation motifs (ITAMs) which, when phosphorylated, promote activation of spleen tyrosine kinase (SYK) and downstream recruitment of a host of secondary molecules including those involved in the phosphoinositide-3 kinase (PI3K) pathway. This series of events is responsible for degranulation of mast cells and can predispose to pathologic mast cell activation when inappropriately upregulated. SYK is recruited to the FcεR1 upon antigen stimulation, and inhibition of this protein has been shown to inhibit mast cell degranulation and production of both lipid mediators and cytokine activity (9). When mast cells from CSU patients with active urticarial disease at the time of blood sample collection were compared to those from healthy human donors, they were unsurprisingly found to release significantly more histamine *in vitro* than their healthy counterparts (10). Yet when these CSU patients were further subdivided into responders vs. non-responders based on their ability to degranulate in response to anti-IgE (with responders showing >10% degranulation activity), SYK levels were shown to be higher in the responder group than in the non-responder group, suggesting that this protein is a major determinant of predilection toward spontaneous degranulation. SYK expression is highly variable among the general population and is thought to correlate with the degree of IgE-mediated degranulation. Intriguingly, the presence of autoantibodies to FcεRIα or IgE do not predispose to upregulation of basophil SYK expression (11).

Negative regulation of mast cell activation occurs through phosphoinositide lipid phosphatases which function as well-described negative regulators of hematopoietic cell activation and proliferation. Src homology 2 (SH2)-containing inositol phosphatases SHIP-1 and SHIP-2 associate with the FcεR1 β subunit and are activated upon stimulation with IgE or antigen (12). It is likely that dysregulation of these pathways that leads to an imbalance of positive signaling plays a pathogenic role in the development of CSU. One study demonstrated that when basophils from highly allergic IgE-positive donors (distinguished as having the ability to mount a response to human-recombinant histamine release factor, a complete stimulus for histamine release) were compared to those from healthy human donors, they contained lower levels of SHIP protein. As such, they demonstrated hyperresponsiveness (i.e., degranulation) in response to stimuli that did not appear to affect IgE-negative basophils (13). Similar results have been shown in mast cells from CSU patients highly sensitive to degranulation, with responders showing significantly lower levels of SHIP protein than both non-responders and healthy human donors (10).

## The Autoimmune Theory of Mast Cell Activation in Chronic Spontaneous Urticaria

While cellular signaling defects may account for some cases of CSU, the autoimmune theory is the more widely accepted hypothesis to explain the inappropriate activation of mast cells and basophils in patients with chronic spontaneous urticaria. Up to 45% of cases of CSU are thought to be autoimmune in etiology. In a sentinel study conducted by Grattan et al., 12 patients with chronic urticaria were subjected to intradermal autologous serum injection (14). Seven of the 12 subjects (of whom six were female) mounted a positive wheal-and-flare reaction to this test, and fewer of these patients described a history of disease exacerbation with application of pressure when compared to patients with a negative injection test. This suggested that these patients with a positive result were less likely to have an inducible urticarial syndrome. Additionally, only one patient described a personal history of atopy, suggesting an alternative etiology for urticaria in the majority of cases. When the same serum was re-injected into the same subjects 1 year later, most of the patients with an initial positive test demonstrated a second positive result, though the same was not necessarily true when fresh serum was injected. In the small number of serum-positive patients who did not mount a second reaction at 1 year, the authors noted that their urticaria had cleared. On the contrary, the two patients who continued to mount a reaction with both original and fresh serum at the 1 year interval were noted to have continued disease activity. Ultimately, the authors concluded that many patients with chronic urticaria contain a “circulating mediator” in their serum which is capable of inducing urticaria. Over the past 30 years, the search has been ongoing to identify the mysterious culprit; multiple theories have since been put forth. However, it was these initial findings that opened the floodgates to the autoimmune theory of chronic spontaneous urticaria.

Though theoretically performed by Grattan et al. (14), the autoimmune etiology of CSU was further supported by formal development of the autologous serum skin test (ASST), an *in vivo* assay of mast cell activation that is induced by intradermal injection of a patient's serum into self. It has now been accepted that nearly 50% of patients with CSU will have a positive test in response to factors present within their own serum within 30 min of injection. Additional groups have suggested that such “factors” are indeed autoantibodies or histamine-releasing factors that are capable of inducing mast cell degranulation. Unfortunately, these data have been somewhat difficult to interpret, as positive ASSTs are not unique to patients with CSU and have been noted in a substantial proportion of patients with allergic or non-allergic rhinitis, multiple drug allergy syndrome, and even in healthy control subjects (15). Furthermore, the positivity of the test has been shown to persist even when CSU patients are in clinical remission, particularly in subjects with autoimmune thyroiditis (16). Interestingly, it has been shown that levels of autoantibodies in CSU do not vary with disease activity which may, in part, explain this finding (17).

Some individuals have suggested that the autologous plasma skin test may be more sensitive than ASST for the diagnosis of CSU (18), though this result has not been reliably demonstrated (19). Since plasma and serum have similar levels of autoantibodies, additional mechanisms beyond autoantibody production have been put forth to explain the pathophysiology of the disease. One such theory states that factors that are uniquely present in plasma may be involved in the development of urticaria, and indeed it was shown that levels of the prothrombin fragment 1+2 (a marker of thrombin generation) were significantly higher in CSU patients than in control subjects (18). This suggests involvement of the clotting cascade in the development of the urticarial reaction. While the role of the coagulation cascade in the pathogenesis of urticaria is beyond the scope of this review, thrombin has been shown to directly increase mast cell degranulation, activate protease-activated receptors on mast cells, and enhance vascular permeability through actions on endothelial cells (20). It should also be noted that autoantibodies against the low affinity IgE receptor FcεRII (CD23) have been demonstrated in a large percentage of CSU patients (21). Anti-CD23 autoantibodies can activate eosinophils to release major basic protein, which in turn can trigger histamine release from mast cells, and basophils. Curiously, in CSU patients eosinophils are also a major cellular source of tissue factor, a protein which promotes activation of the extracellular coagulation cascade and generation of thrombin (22).

It has also been speculated that activated lymphocytes may play a role in the pathogenic activation of mast cells. Indeed it has been demonstrated that mast cells release inflammatory mediators including TNF-α upon direct contact with activated T cells (23). This TNF-α release is responsible for upregulation of several mast cell genes, among which includes matrix metalloproteinase 9 (MMP9) and tissue inhibitor of metalloproteinase 1 (TIMP1). Intriguingly, MMP9 and TIMP-1 have both been found at higher levels in the plasma of patients with chronic urticaria, and levels of MMP-9 may correlate with disease severity (24).

### Chronic Spontaneous Urticaria Is Associated With Development of Autoantibodies to IgE and the High Affinity IgE Receptor

Circulating mediators with the potential to induce wheal-and-flare reactions in nearly half of all patients with CSU have been demonstrated in response to ASST. The identify of those factors became apparent in the early 1990s. One landmark study demonstrated that antibodies present in the serum of patients with chronic spontaneous urticaria (but not in patients with dermatographism or pressure urticaria) were able to elicit histamine release from healthy donor peripheral blood leukocytes with similar kinetics as anti-IgE antibodies (25). Intriguingly, the authors noted that preincubation of basophils with either anti-IgE or chronic urticarial serum abolished subsequent histamine release upon incubation with urticarial serum or anti-IgE, suggesting that both anti-IgE and chronic urticarial serum stimulate degranulation via a similar mechanism. Removing surface-bound IgE from leukocytes prior to incubation with urticarial serum or anti-IgE reduced histamine release. These important results suggested for the first time that histamine-releasing autoantibodies present in the serum of patients with chronic urticaria act by cross-linking cell surface IgE receptors.

In addition to the aforementioned findings, the authors astutely noted that histamine release could be elucidated when chronic urticarial serum was incubated with basophils from donors with very low serum IgE concentrations but not with anti-IgE (25). These data implied that non-IgE-dependent histamine releasing factors are present in the serum of patients with CSU. Shortly after the publication of this initial study, the same group determined the presence of IgG antibodies against the α subunit of the high-affinity FcεR1 in a group of patients with CSU (26). In this subset of patients, IgG anti-FcεR1α pathologically induced histamine release irrespective of the degree of IgE sensitization of the basophils. As proof of concept, histamine release was effectively neutralized in a concentration-dependent manner by preincubating donor basophils with soluble fragment of FcεR1α prior to the addition of purified IgG from sera of patients with CSU.

The concept that circulating IgG antibodies against IgE and the high-affinity IgE receptor FcεR1 likely contribute to the pathogenesis of CSU has since become widely accepted. Approximately 40% of patients with CSU have circulating antibodies to one of these targets (27) with a higher frequency of positivity in CSU patients who are ASST positive (28). Anti-FcεRI antibodies are thought to be the more common of the two. FcεRI is found on the surface of both dermal mast cells and basophils, and autoantibodies to this receptor can provoke chronic stimulation and degranulation of these cells in an IgE-independent fashion (2). On the contrary, IgG-anti IgE antibodies may bind to and crosslink receptor-bound IgE on the surface of mast cells and basophils, thus leading to activation and degranulation of these cells. As is the case with many autoimmune conditions, the presence of autoantibodies does not necessarily result in a disease phenotype. The presence of FcεR1α autoantibodies have been noted in the sera of patients with other autoimmune skin conditions and even in healthy subjects, though these have not been shown to display pronounced histamine-releasing activity in individuals without CSU (29). The authors attribute this difference to the fact that anti-FcεR1α antibodies tend to be of the complement-fixing IgG1 and IgG3 subtypes in patients with chronic urticaria but of IgG2 and IgG4 subtypes in patients with other inflammatory skin conditions. Other groups have shown that *in vitro* basophil activation and subsequent histamine-releasing activity fails to correlate with the presence of autoantibodies to FcεR1α even among patients with chronic urticarial (27).

The presence of autoantibodies to IgE and to FcεR1α infers the presence of antigen-specific lymphocytes in individuals with chronic urticarial disease. FcεR1α-specific T lymphocytes are detectable in a large percentage of patients with CSU and these cells more typically adopt a Th1 cytokine profile with the largest percentage being INF-γ secretors (30). Intriguingly, the relationship between INF-γ and autoantibody responses to FcεR1α has been found to be inversely related, similar to that which has been observed for T cell and autoantibody reactivity to glutamic acid decarboxylase antigen in individuals at risk for type 1 diabetes mellitus (31). It remains unclear how T cell reactivity vs. antibody reactivity to FcεR1α affects pathogenesis of CSU. However, it has been demonstrated that markers of T cell activation are directly proportional to markers of mast cell degranulation in chronic urticaria patients, particularly in patients known to have positive antibodies against FcεR1 (32). Further evidence for the involvement of T lymphocytes in the pathogenesis of CSU stems from observed variations in protein tyrosine phosphatase 22 (PTPN22) in patients with CSU (33). PTPN22 is a strong susceptibility gene for a variety of autoimmune disorders and encodes lymphoid specific tyrosine phosphatase (Lyp), which normally serves as an inhibitor of T cell activation.

An increased frequency of the HLA-DR4 allele has been found in patients with CSU (34). Intriguingly, the HLA-DR4 has been found at an increased frequency in a variety of other autoimmune disorders including rheumatoid arthritis, type 1 diabetes mellitus, and multiple sclerosis. Patients with autoimmunological subtypes of CSU have been noted to have a particularly high likelihood of carrying this HLA class II allele (35). However, these data have not been replicable across a wide spectrum of populations, with other studies noting increased frequencies of HLA-DR9 (30) and HLADR12 (30, 36) among patients with CSU. Heterogeneity in allelic association with this disease likely indicates that FcεR1α contains a number of different epitopes which act as targets of autoreactive T lymphocytes.

### The Overlap Between Chronic Spontaneous Urticaria and Other Autoimmune Diseases

The concept of “overlapping autoimmune diseases” suggests that disorders which are autoimmune in nature occur at increased frequency in patients with known autoimmune disease. Numerous autoimmune conditions including systemic lupus erythematosus, polymyositis, dermatomyositis, and rheumatoid arthritis have been associated with chronic urticaria (2).

One large population study of over 12,000 subjects derived from a large health maintenance organization in Israel determined that female patients with CSU demonstrate a significantly higher incidence of rheumatoid arthritis, Sjögren syndrome, celiac disease, type I diabetes, and systemic lupus erythematosus than patients without CSU (37). While men also demonstrated higher odds of having these autoimmune conditions when compared with control subjects, these numbers did not reach statistical significance. When further investigation into serologic markers of autoimmune disease was performed, it was determined that patients with CSU as compared to control subjects had significantly higher levels of anti-thyroid peroxidase (anti-TPO) antibodies, antinuclear antibodies (ANA), antithyroglobulin (i.e., antimicrosomal) antibodies, rheumatoid factor, anti-transglutaminase IgA antibodies, and anti-parietal cell antibodies with anti-dsDNA, and anti-cardiolipin antibodies trending toward significance. Moreover, the mean platelet volume (MPV) was noted to be abnormally high in 29% of CSU patients and only in 1% of control subjects. Elevations in MPV occur when the body produces platelets at a more rapid rate and tend to correlate with levels of systemic inflammation.

As this study was the first of its kind to examine the relationship between CSU and other autoimmune diseases, it has shed some light on some intriguing commonalities between these two conditions. As is the case with most autoimmune conditions, CSU tends to affect women more commonly than it does men, and women with CSU tend to have much higher odds of developing other autoimmune conditions vs. men. This sex difference is also mirrored by the observation that both CSU and autoimmune diseases on the whole tend to occur more commonly in young adulthood as opposed to in older, post-menopausal adults. Additionally, the high prevalence of the aforementioned autoimmune conditions in CSU patients at much higher frequencies than occurs in the general population adds more strength to the theory that the underlying pathology of CSU is autoimmune in nature. When overlapping autoimmune conditions did occur, they were frequently diagnosed within the first 10 years after onset of CSU and quite commonly within the first 6 months. A recently conducted systematic review of autoimmune comorbidities in individuals with CSU also noted that organ-specific autoimmune disorders are more common than systemic autoimmune disorders in patients with urticaria, with endocrine, hematologic and skin disorders being among the most common (38). The reasons for this discrepancy remain largely unclear.

The common pathogenic mechanism between these conditions is the presence of autoantibodies on a background of chronic inflammation. However, autoimmune disorders are incredibly heterogenous in nature and thus it is difficult to extrapolate whether the link between chronic urticaria and the aforementioned conditions truly stems from a common pathologic tie or is merely reflective of detection bias. If a true connection does exist, one would imagine that CSU would be found at a higher frequency in patients with established autoimmune disease, but this link has been difficult to definitively make. For example, the prevalence of CSU in patients with SLE ranges from 0 to 22% depending on the individual study (39).

### Autoallergy in Chronic Spontaneous Urticaria

With regards to the idea of overlapping autoimmune diseases, the well-established link between chronic urticaria and autoimmune thyroid disease deserves particular mention. Even among euthyroid subjects, many patients with CSU have detectable levels of antibodies against thyroglobulin or microsomal-derived antigen (40). Furthermore, an increased prevalence of clinical hypothyroidism (i.e., Hashimoto's thyroiditis) as well as hyperthyroidism has been found among patients with CSU, with one study estimating a 23 times and seven times greater odds for hypothyroidism in female and male patients with chronic urticaria compared to control subjects, respectively (37). In 80% of these cases, the diagnosis of thyroid disease was made within 10 years, of the diagnosis of urticaria. The occurrence of IgG anti-thyroid antibodies in patients with CSU documented in studies where more than 100 patients were enrolled was noted to be anywhere from 3.7 to 37.1% with two-thirds reporting increased anti-thyroid antibody levels in >10% of patients (41).

Patients with CSU also demonstrate higher levels of IgE anti-thyroid peroxidase (anti-TPO) antibodies relative to healthy controls, though this distribution was found to be bimodal with 39% of CSU patients exhibiting IgE anti-TPO levels similar to control subjects (IgE anti-TPO^low^) (42). It is theorized that autoallergic mast cell activation may contribute to the pathophysiology in CSU patients with detectable levels of IgE anti-TPO. IgE has a well-established role in the defense against helminthic infections and in recognition of exogenous allergens, but it was not until very recently that its potential role in autoimmunity has emerged. The term “autoallergy” refers to a type I, IgE-mediated hypersensitivity reaction against self-antigens which can, in turn, promote degranulation of basophils and mast cells. It was first put forth by Rorsman et al. as an explanation for urticaria-induced basopenia (43). Rorsman theorized that unlike in physical causes of urticaria, antigen-antibody interactions in non-physical causes of urticaria are likely to occur in both the skin and within the circulation (44). Autoallergic mast cell activation has been shown to occur in a variety of skin disorders including atopic dermatitis (45, 46) and bullous pemphigoid (47). In such disorders, IgE directed against skin antigens may bind to these cognate antigens and activate mast cells residing within the skin. On the contrary, TPO can be released from the thyroid into circulation, where it is free to bind to the surface of FcεR1-expressing cells. This extracutaneous nature of TPO may be one reason why the manifestations of CSU are not simply limited to the skin as they are in many other autoimmune skin disorders. Indeed it has been shown that anti-IgE TPO antibodies have the ability to induce basophil degranulation *in vitro* in the presence of TPO antigen and likely play a role in the pathogenesis of CSU (48). Recent findings have demonstrated that IgE anti-TPO antibodies are present at higher frequency and amounts in patients with CSU and have greater potential to induce TPO-mediated skin reactions in these subjects vs. in healthy controls (49). These results were validated by increased upregulation of basophil activation markers in CSU subjects upon exposure to TPO and ability of anti-TPO IgE to induce positive skin reactions upon passive transfer of this antibody from a patient with CSU to the skin of a healthy subject. In addition to IgE anti-TPO antibodies, IgE anti-dsDNA antibodies have also been noted at higher frequency in patients with CSU (50). However, no significant difference in IgE anti-dsDNA levels were observed between ASST-positive vs. ASST-negative patients, which suggests that these antibodies may be correlated with but are not likely to be involved in the pathogenesis of CSU.

IgE-mediated autoimmunity is becoming increasingly recognized as a possible contributor to the pathogenesis of a variety of systemic conditions including systemic lupus erythematosus and rheumatoid arthritis (51). A multicenter study in patients with SLE showed over half of all subjects had detectable levels of IgE against at least one common nuclear autoantigen (dsDNA, SS-A, SS-B, Sm) (52). This frequency increased during active disease and was strongly associated with the presence of active nephritis. There is still much to learn about the fields of autoallergy and IgE-mediated autoimmunity, which currently remain in their infancy, but it is likely that this will reveal a host of novel, targetable autoantigens. Over 200 IgE autoantigens were recently demonstrated in CSU subjects that were not present in healthy controls, among which included IL-24, which were detected in all patients with CSU (53). Further *in vitro* analyses demonstrated that IL-24 contributes to histamine release from human mast cells sensitized with IgE from CSU but not control subjects and that IgE-anti-IL-24 levels have reasonable predictive value for disease activity. The questions of why and how IgE autoantibodies develop and to what degree they contribute to CSU pathogenesis when compared to IgG autoantibodies has yet to be determined and is likely to be the focus of many future studies in this field.

## Treatment of Chronic Spontaneous Urticaria

The primary treatment for chronic urticaria is to address the underlying pathology whenever possible and prevent mast cell activation. In any patient with chronic urticaria, elimination of potential triggers including drugs which can cause non-allergic hypersensitivity reactions (most commonly NSAIDs) should first be undertaken (5) Multiple guidelines have been put forth for management of CSU, though the EAACI/GA^2^LEN/EDF/WAO remain the most popular among practicing clinicians (5). If cases where no triggers can be identified, antihistamines (particularly the modern 2nd-generation antihistamines) are recommended as the mainstay pharmacologic treatment modalities for this condition. In patients who are poorly responsive to antihistamine therapy, it is recommended that the dosage be increased up to four times the normal limit prior to initiating a new agent. In cases of severe urticaria not adequately controlled with antihistamines alone, the EAACI/GA^2^LEN/EDF/WAO guidelines have recommended the addition of anti-IgE therapy, with cyclosporine being reserved for the most refractory cases of CSU. While short courses of prednisone do have a role in acute exacerbations of CSU, there is a strong recommendation against the use of long-term oral steroids given risk for adverse effects.

### Evaluating the Use of Omalizumab for the Treatment of Chronic Spontaneous Urticaria

Omalizumab is an IgG monoclonal antibody against the Fc portion of the IgE antibody and prevents free IgE from binding to high-affinity FcεR1 receptors on mast cells and basophils. The first multicenter, placebo-controlled study evaluating omalizumab use in CSU examined efficacy of this therapy in subjects with IgE autoantibodies and found that 70% of omalizumab-treated patients who were otherwise refractory to standard antihistamine therapy were completely protected against wheal development (vs. 4.5% of placebo-treated subjects) (54). Another phase three study demonstrated that omalizumab given in three subcutaneous doses of either 150 or 300 mg over a 12 week period significantly reduced symptoms in antihistamine-refractory CSU patients without rebound of symptoms following discontinuation of the medication (55). Lack of relapse after discontinuation and very good safety profile have made omalizumab suitable for long-term therapy in patients with CSU, though cost still limits its use in many circumstances.

The mechanism by which omalizumab benefits patients with CSU has yet to be fully elucidated but the aforementioned results strongly argue for the contribution of IgE autoantibodies in the pathogenesis of CSU with rapid neutralization of these antibodies being an effective component of therapy. While the exact mechanisms by which omalizumab treatment benefits patients with CSU remain unclear, clearance of IgE autoantibodies is certainly likely to be of relevance. It has been well-established that omalizumab rapidly reduces levels of free IgE, which promotes downregulation of FcεR1 on basophils within the span of weeks and on mast cells within the span of months (56). The reason for this is because FcεR1 is effectively degraded when it is not stabilized by IgE binding (56, 57). Additionally, omalizumab has been shown to improve the genetic signature of lesional skin in patients with CSU to reflect non-lesional skin signatures by downregulating expression of genes involved in mast cell and leukocyte infiltration (FCER1G, C3AR1, CD93, S100A8), oxidative stress, vascularization (CYR61), and skin repair (KRT6A, KRT16) (56). Notably, non-responders to omalizumab do not demonstrate these genetic alterations. Basophils are also thought to be recruited to the urticarial lesions of patients with active CSU (58). *Post hoc* analysis of randomized clinical trial data have demonstrated that omalizumab increases peripheral blood basophil counts by likely reducing recruitment of these cells to the skin (59) and may also help to regulate defective basophil IgE receptor pathways (57). Clinical trials are currently underway to better characterize the effects of omalizumab on basophil responses.

Treatment with omalizumab has been shown to decrease levels of both FcεR1- and IgE- positive skin cells in skin of patients with CSU (60). In theory, omalizumab may reduce the autoimmune effects of self-antigen by mediating this decrease in pathogenic IgE levels and/or IgE receptors on the mast cell surface. Recently, a great deal of attention has been placed on the utility of IgE levels for predicting responses to omalizumab. Patients who have lower IgE levels prior to receiving omalizumab and lower IgE levels 4 weeks after undergoing treatment tend to respond less well to this therapy than those with higher baseline and post-treatment IgE levels (61), suggesting that these laboratory tests may help to guide management of CSU patients being considered for second- and third-line treatment options (61, 62). Interestingly, total IgE levels have been shown to positively correlate with basophil FcεR1 expression, the latter of which tends to be higher in those who respond quickly to omalizumab therapy (62). Despite our poor knowledge of the precise role of basophils in CSU pathophysiology, it is becoming clear that the time to effect for omalizumab in CSU appears to correlate with the expected time for reduction of FcεR1 on basophils (54) as opposed to on mast cells. Similar results were noted in prior studies examining use of omalizumab for cat allergy, which demonstrated that reduction of nasal symptoms correlated directly with a reduction in basophil responsiveness as opposed to mast cell responsiveness (63).

The question of how omalizumab benefits patients whose disease is mediated by a type I hypersensitivity reaction (autoallergy) vs. a type II hypersensitivity reaction (autoimmunity) is still largely unclear, however, it does appear that patients with autoallergy experience faster response times when treated with omalizumab. In one study, subjects refractory to standard antihistamine therapy with IgE anti-TPO antibodies were randomized to receive omalizumab in 2 or 4 week intervals for a span of 24 weeks. Patients with anti-IgE TPO antibodies experienced early responses to omalizumab, suggesting that rapid neutralization of IgE is the major mechanism by which omalizumab benefits this particular cohort (54). On the contrary, subjects who display a slow response to omalizumab are thought to have IgG antibodies against FcεRI since downregulation of this receptor occurs only after free IgE is first complexed by the drug (64). The authors validated this hypothesis by demonstrating a high correlation between length of time to the onset of omalizumab efficacy and positive basophil histamine release activity, with the latter predicting slower response times to treatment (64). Basophil activation, which is more specific for the detection of histamine-releasing autoantibodies in CSU patients than the ASST, may thus be a useful test in assessing patient responsiveness to omalizumab (64). As such, patients with refractory CSU may benefit from longer, more “personalized” courses of omalizumab (65). Further investigation on the mechanisms by which omalizumab benefits patients with CSU is certainly in need as are biomarkers to predict the efficacy of responsiveness and likelihood of relapse among patients with various subtypes of CSU.

## Conclusion

Chronic spontaneous urticaria is a common and complex disorder that occurs in the absence of any identifiable provoking factor. While there are many aspects regarding CSU that have yet to be understood, it is becoming increasingly clear that both autoimmunity (IgG-mediated disease) and autoallergy (IgE-mediated disease) can contribute to the pathogenesis of this disorder and predispose subjects to the development of additional autoimmune diseases. Subjects with IgE autoantibody-mediated CSU appear to have a faster onset of improvement in response to omalizumab than those with IgG-mediated disease due to the unique mechanisms by which this drug sequentially affects IgE levels and FcεR1 status. Further investigation is required to determine how the presence of unique autoantibodies can predict the disease course and comorbidities associated with various subtypes of CSU as well as overall responsiveness to therapy.

## Author Contributions

SB and ASM conceptualized the topic of the review article and wrote the review. SA and ASM supervised the writing of this review.

### Conflict of Interest Statement

The authors declare that the research was conducted in the absence of any commercial or financial relationships that could be construed as a potential conflict of interest.
